# Targeting Metabolic Dysfunction in Parkinson’s Disease: The Role of GLP-1 Agonists in Body Weight Regulation and Neuroprotection

**DOI:** 10.1007/s11892-025-01606-1

**Published:** 2025-09-26

**Authors:** Iciar Aviles-Olmos, Christian Espinoza-Vinces, Leyre Rogel Portugal, María Rosario Luquin

**Affiliations:** 1 Department of Neurology, Clínica Universidad de Navarra. Pamplona, Av. de Pío XII, 36, Pamplona, 31008 Navarra Spain; 2 Navarra Institute for Health Research (IdiSNA), Pamplona, Spain; 3 Faculty of Medicine, Universidad de Navarra, Pamplona, Spain

**Keywords:** Parkinson’s disease, GLP-1 receptor agonists, Metabolism, Body weight, Neuroprotection, Diabetes, Obesity

## Abstract

**Purpose of Review:**

This review explores the role of GLP-1 receptor agonists (GLP-1 RAs) in addressing metabolic dysfunction and neurodegeneration in Parkinson’s disease (PD), focusing on body weight regulation and neuroprotection.

**Recent Findings:**

GLP-1 RAs modulate insulin signaling, reduce neuroinflammation and oxidative stress, and improve mitochondrial functional mechanisms linked to neuroprotection. Clinical trials show modest but sustained improvements in motor symptoms and suggest benefits in cognition, mood, and apathy. While GLP-1 RAs induce weight loss in diabetes, their metabolic impact in normoglycaemic PD patients appears limited. However, individuals with obesity or insulin resistance may experience enhanced clinical and cognitive outcomes.

**Summary:**

GLP-1 RAs offer a multifaceted therapeutic strategy in PD, targeting both central neurodegenerative processes and peripheral metabolic dysfunction. Their potential for disease modification and symptom relief, particularly in specific phenotypes, supports their further exploration as part of a personalized treatment approach.

**Supplementary Information:**

The online version contains supplementary material available at 10.1007/s11892-025-01606-1.

## Introduction

Parkinson’s disease (PD) is a progressive neurodegenerative disorder characterized by three main motor symptoms: resting tremor, rigidity and bradykinesia. However, non-motor symptoms, such as cognitive impairment and autonomic dysfunction, potentially linked to neuroendocrine and metabolic dysfunction, are now widely recognized as important features that extend beyond the loss of dopaminergic neurons [1].

The pathophysiological processes in PD, including oxidative stress, protein misfolding, mitochondrial dysfunction and neuroinflammation, overlap with those observed in type 2 diabetes (T2D) [2–5]. Central insulin resistance contributes to neurodegeneration by promoting mitochondrial failure, protein aggregation, and apoptosis. Epidemiological studies have linked T2D to an increased risk of PD, as well as to more severe motor and cognitive symptoms in individuals with both conditions [6–8]. These associations have sparked growing interest in repurposing antidiabetic drugs, particularly those that target insulin signaling, as potential disease-modifying therapies for PD.

Glucagon-like peptide-1 (GLP-1) is an incretin hormone that enhances insulin secretion and exhibits anti-inflammatory, antioxidant and anti-apoptotic properties [9]. GLP-1 receptors are not only expressed in the pancreas and gastrointestinal tract, but also in the brain, where they influence neuronal survival and metabolism [9]. Notably, GLP-1 receptor agonists (GLP-1 RAs) can cross the blood-brain barrier, and in preclinical PD models, GLP-1 RAs have been shown to improve motor function and provide neuroprotection [10, 11]. Based on these findings, clinical trials, particularly those involving Exenatide and Lixisenatide, have explored their potential to modify the course of PD, however, recent findings have been less promising than anticipated.

GLP-1 RAs act via both peripheral and central mechanisms. Peripherally, they reduce insulin resistance, slow gastric emptying and promote weight loss, which is beneficial for T2D but potentially problematic in PD [12, 13]. This is due to a complex, sometimes conflicting relationship between obesity and PD [14, 15].

In advanced PD, weight loss is common, driven by anorexia, dysautonomia, tremor and dyskinesia-related energy expenditure, and sarcopenia [16, 17]. This unintentional weight loss is associated with frailty, falls, and cognitive decline.

In early-stage PD, findings vary across studies. A large cross-sectional study found that a body shape index, which reflects visceral fat, was positively correlated with PD risk, particularly in men and individuals under 60 years old [18]. Conversely, Mendelian randomization studies suggest higher lifetime Body Mass Index may be protective, and underweight or diabetic individuals have a greater PD risk [19, 20].

Considering this biphasic weight pattern in PD, the dual action of GLP-1 RAs, reducing insulin resistance while inducing weight loss, raises important considerations regarding their long-term metabolic effects in PD patients with varying phenotypes and nutritional profiles.

This review examines the neuroprotective potential and metabolic effects of GLP-1 RAs in PD, with particular emphasis on their impact on weight regulation, underlying mechanisms, clinical outcomes, and therapeutic prospects.

## Methods

### Study Identification

This systematic review was conducted following the Preferred Reporting Items for Systematic Reviews and Meta-Analyses (PRISMA) guidelines [21]. A comprehensive Literature search was performed on May 8th, 2025, across the following electronic databases: PubMed, Cochrane Library, Scopus, and Web of Science. The search process was independently carried out by two reviewers (IAO and CEV). The following search string was used:

- #1"Parkinson Disease"[mh] OR Parkinson* [tw]) AND
- *#2"Glucagon-Like Peptide 1"[mh] OR"GLP-1 receptor agonists"[tw] OR"glucagon-like peptide-1"[tw] OR GLP-1 [tw] OR liraglutide [tw] OR exenatide [tw] OR dulaglutide [tw] OR semaglutide [tw] OR lixisenatide [tw]) AND*
- *#3"Metabolism"[mh] OR metabolism [tw] OR"Body Weight"[mh] OR"body weight"[tw] OR"Obesity"[mh] OR obesity [tw] OR"Diabetes Mellitus"[mh] OR diabetes [tw] OR neuroprotection [tw])*
  *AND English [la] AND ("2000"[dp]:"2025"[dp])*

### Study Selection

The following authors, IAO and CEV, independently reviewed the titles and abstracts of all identified articles. Any study that appeared to meet the predefined eligibility criteria—outlined in Table 1 and focused on the role of GLP-1 agonists in body weight regulation and neuroprotection, was selected for further evaluation and full-text review.

**Table 1:** Eligibility criteria of the articles

Inclusion criteria	● Human and animal studies ● Peer-reviewed full-text articles only ● Published between January 2000 and April 2025 ● Research addressing the use or application of GLP-1 receptor agonists in Parkinson’s disease, particularly in relation to metabolic dysfunction, body weight regulation, or neuroprotection ● Articles written in English
Exclusion criteria	● Articles not available in full text ● Publications in languages other than English. ● Systematic reviews, conference abstracts, editorials, commentaries, or letters without original data. ● Studies not specifying Parkinson’s disease diagnosis.

**Fig. 1:**
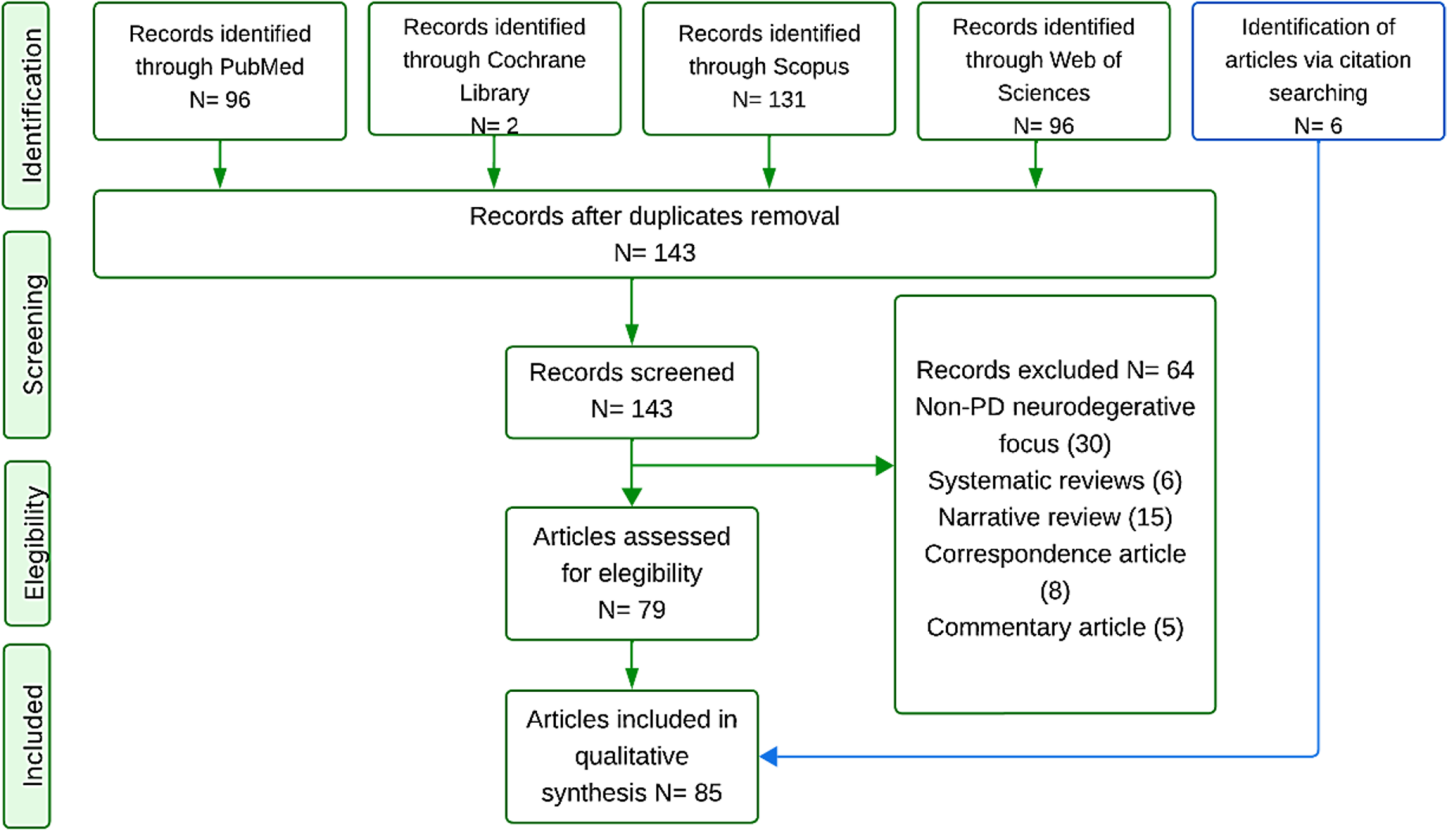
Article selection process

Discrepancies between the two reviewers were addressed through discussion and consensus. In addition to the primary search strategy covering 2000–2025, manual reference screening of key articles was performed. Following PRISMA guidelines, three seminal articles published prior to 2000 were also included due to their foundational relevance to the pathophysiology of Parkinson’s disease and metabolic regulation (Fig. 1).

### GLP-1 Agonists and Body Weight in Parkinson’s Disease

The regulation of weight in patients suffering from PD is a complicated matter, involving a combination of neurodegenerative, metabolic, and gastrointestinal factors. Even in early PD, unintentional weight loss is common, and this has been linked to sarcopenia, frailty, and accelerated disease progression [22–24]. While GLP-1 RAs are well-established in the treatment of T2D, their metabolic effects in PD may benefit from a more detailed and context-specific assessment, particularly regarding body mass index (BMI), fat mass, and appetite.

### Epidemiology

Epidemiological studies have increasingly explored the relationship between metabolic dysfunction and the progression of PD. Large-scale, population-based cohort studies have demonstrated an association between T2D and an increased risk of developing PD, with hazard ratios ranging from 1.2 to 1.5, depending on the study design and population [6, 25–28]. Furthermore, individuals with insulin resistance or obesity appear to experience a faster decline in motor and cognitive function once PD is established [7, 27].

Retrospective pharmacoepidemiologic studies have investigated whether treatment with GLP-1 RAs can influence PD incidence or trajectory. One study using Danish registries reported a reduced risk of PD among diabetic patients treated with GLP-1 RAs compared to those receiving other antidiabetic medications [8]. Similarly, a UK-based population study using real-world clinical data found that exposure to exenatide was associated with a lower probability of a PD diagnosis over time [29]. For older adults with type 2 diabetes, starting GLP-1 receptor agonists was found to significantly reduce the risk of developing PD compared to dipeptidyl peptidase-4 inhibitors [30].

### Changes in BMI and Fat Mass

GLP-1 RAs (e.g., liraglutide, semaglutide, exenatide) consistently reduce BMI and fat mass in T2D patients, primarily through appetite suppression and delayed gastric emptying [12, 13, 31]. This weight loss is generally advantageous for overweight diabetic patients.

However, data in PD populations are Limited and heterogeneous. In the Exenatide-PD trial, which involved non-diabetic individuals, no significant reduction in BMI was reported after 48 weeks of exenatide treatment [32]. Similarly, an earlier open-label trial by the same group found minimal weight change over 12 months [33]. This may reflect that GLP-1 RAs may not exert their typical weight-lowering effects in normoglycaemic PD patients. In the recent exenatide trial [34], the difference in weight loss between the exenatide and placebo groups was 0.5 kg, which is lower than the 2.0 kg difference observed in a previous randomised trial [32]. There were no differences between groups according to their BMI at baseline.

### Appetite and Nutritional Intake

Loss of appetite is a known side effect of GLP-1 RAs and occurs regardless of diabetic status [12, 35]. In PD, where anorexia and delayed gastric emptying are already common due to autonomic dysfunction, this may exacerbate nutritional deficiencies. Although nausea and early satiety are manageable in younger T2D populations, older PD patients may be more vulnerable, particularly those already underweight or with impaired oral intake [16, 17].

Qualitative reports from PD trials have described reduced appetite as a common adverse effect, but quantitative data on caloric intake or nutritional biomarkers remain limited in the literature [32–34].

### Diabetic Vs. Non-Diabetic Populations

The metabolic response to GLP-1 RAs varies significantly between diabetic and non-diabetic patients with PD. Diabetics, particularly those who are obese or insulin resistant, are more likely to experience reductions in BMI and fat mass. This is consistent with the well-established mechanisms of action of GLP-1 [36–38]. These effects could improve cardiovascular and metabolic outcomes, making GLP-1 RAs more beneficial for PD patients with metabolic comorbidities.

By comparison, non-diabetic PD patients show a diminished weight effect, with most studies reporting a stable BMI. This may be due to preserved endogenous GLP-1 activity, lower baseline insulin resistance, or PD-related hypothalamic dysfunction affecting appetite signaling. Furthermore, weight loss is likely to remain a concern in non-diabetic patients, particularly if they are already frail or undernourished, and especially in the context of the semaglutide trial, given its enhanced efficacy in promoting weight reduction [39].

### GLP-1 Agonists and Mechanistic Rationale

#### Preclinical Data: Inflammation, Oxidative Stress, Mitochondrial Function

Emerging research in animal models of PD suggests that GLP-1RA may hold promise in addressing the underlying mechanism and symptoms associated with PD. GLP-1 is a peptide hormone that easily crosses the blood-brain barrier, making GLP-1RAs good candidates for treating several neurodegenerative diseases like PD and Alzheimer’s disease, brain injury, and stroke [40]. GLP-1Rs are highly expressed in the frontal cortex, hypothalamus, thalamus, hippocampus, cerebellum, and substantia nigra pars compacta (SNpc) [9, 41]. GLP-1R activation leads to an increase of intracellular cyclic adenosine monophosphate (cAMP), which activates protein kinase A (PKA) and phosphoinositide 3-kinase (PI3K) with a subsequent activation of some downstream signaling pathways associated with cell survival [42].

In PD, the essential degeneration process is influenced by several pathophysiological factors, including mitochondrial dysfunction, increased oxidative stress, inflammation, neurotrophic factor deficiency, impairment of the proteasome-autophagia process, and generation of α-synuclein oligomers that form the Lewy bodies [2–5, 43, 44].

In the MitoPark PD mice, in which mitochondrial function is impaired in the dopaminergic neurons, PT320, a sustained release formulation of the GLP-1RA exenatide, reduced ROS production and improved tyrosine hydroxylase expression. It also preserved mitochondrial function and morphology and enhanced motor activity. These findings support a neuroprotective effect of GLP-1 agonists, likely mediated by mitochondrial preservation [45]. Additionally, in MPTP-mice, liraglutide preserved the number of dopaminergic cells, mitochondrial biogenesis and mitochondrial dynamics. This effect seems to be mediated by activation of the AMPK/PGC-1a (peroxisome proliferation-activated receptor -γ-coactivator 1a) pathway since lentivirus-induced downregulation of PGC-1α reversed the neuroprotective effect [46, 47]. Similar results have been reported in the rotenone and 6-OHDA PD model with different GLP-1RA agonists. In these PD models, sitagliptin and liraglutide improved motor activity, preserved nigral cell death, increased GDNF levels, but, interestingly, they markedly decreased proinflammatory cytokines, probably by inhibiting the AMPK/NF-kB signaling pathway.

Moreover, expression of the pro-apoptotic protein Bax was reduced while Bcl2 expression increased, revealing that GLP-1RA can inhibit the apoptotic pathway. All these data indicate that GLP-1RA might prevent nigral cells from the neurodegenerative process associated with PD by acting on different pathways [10, 48]. More recently, the probiotic Lactobacillus plantarum SG5 has been shown to ameliorate motor deficits, neuronal death and the decreased colonic GLP-1 created by MPPT, indicating that the neuroprotective effect of SG5 likely involves modulation of the gut microbiota and, significantly, the GLP-1/PCG 1α pathway [49].

GLP-1R stimulation may exert a neuroprotective effect by activation of BDNF and GDNF pathways. 2-month treatment with exenatide in mice promotes the enhancement of long-term memory performances along with the activation of the BDNF-TrkB neurotrophic axis and inhibits apoptosis by decreasing p75NTR-mediated signaling [50, 51]. Moreover, in 6-OHDA lesioned rats, treatment with dual GLP-1/GIP analogues attenuated dopaminergic cells and motor deficits, while levels of the growth factor GDNF and pAkt/CREB cell signaling were enhanced [52].

Interestingly enough, in 6-OHDA rats, the dual GLP-1/GIP agonist (DA5-CH), in addition to promoting the survival of dopaminergic neurons, was able to reduce the levels of monomer and oligomers of α-synuclein, thus providing a wider neuroprotective effect [53].

In addition to the improvement of motor deficits created by dopaminergic degeneration, GLP-1 RAs like Sitagliptin and Liraglutide attenuated levodopa-induced dyskinesias in rotenone-lesioned rats, probably due to the ability of GLP-1 RAs to restore the striatal dopaminergic tone [10].

### Spectrum of GLP-1 Effects in Parkinson’s Disease

#### Clinical Improvement

GLP-1 RAs have emerged as promising therapeutic candidates in PD, particularly due to their dual action on metabolic and neurodegenerative pathways. Clinically, early trials have demonstrated meaningful benefits. In a single-blind trial, 12-month exenatide treatment improved motor and cognitive scores compared to controls (MDS-UPDRS difference: +4.9 points; *p* = 0.037) [54, 55].

Subsequent randomized, placebo-controlled trials have reinforced these findings. A 48-week trial of weekly exenatide (2 mg) showed significant motor improvements (− 3.5 MDS-UPDRS III points; *p* = 0.0318), which were maintained after a 12-week washout [32].

Similarly, a recent phase 2 trial of Lixisenatide in early PD showed a stable motor score over 12 months (− 0.04 points), contrasting with a decline in the placebo group (+ 3.04 points), resulting in an adjusted mean difference of 3.08 points (*p* = 0.007). These effects were sustained off medication, further supporting a disease-modifying role [56].

In contrast, a 54-week trial of liraglutide did not show significant motor improvement (MDS-UPDRS III), but daily living activities (MDS-UPDRS II) improved significantly, suggesting functional gains without direct motor benefit [57]. This contrasts with exenatide and lixisenatide, which showed clearer motor effects, possibly due to differences in drug properties, trial design, or patient characteristics [54, 56].

However, not all trials have been successful. NLY01, a long-acting pegylated exenatide form failed to show efficacy in a 36-week phase 2 study despite good tolerability [58]. Likewise, a recent 96-week phase 3 trial of extended-release exenatide in early-to-moderate PD did not meet its primary motor endpoint, though the drug was safe and well tolerated [34].

#### Impact on Dyskinesias and Motor Fluctuations

Regarding dyskinesias, different trials did not demonstrate significant differences between groups in dyskinesia-related outcomes or in motor fluctuations. In the first proof-of-concept trial, exenatide was associated with an increase in the dyskinesia rating scale at both 12- and 14-month time points [54]. In a phase 3, multicenter, double-blind, placebo-controlled trial of once weekly exenatide versus placebo, no significant between-group differences were observed on the Unified Dyskinesia Rating Scale [34]. Similarly, no differences in MDS-UPDRS part IV scores were found in the lixisenatide trial [56].

#### Impact on Non-Motor Symptoms

GLP-1 RAs have shown encouraging potential in addressing non-motor symptoms in PD, which are major contributors to disability and reduced quality of Life. Previous studies have demonstrated sustained improvements in cognitive and mood outcomes among exenatide-treated patients, with a significant 5.3-point advantage on the Mattis Dementia Rating Scale-2 (DRS-2; *p* = 0.006) and favorable scores on the Montgomery-Åsberg Depression Rating Scale (MADRS), persisting 12 months post-treatment, suggesting possible disease-modifying effects [54, 55].

Athauda et al. found significant reductions in apathy and depressive symptoms within the same cohort, with lower NMSS mood/apathy scores (*p* = 0.026), better MDS-UPDRS Part I mood item scores (*p* = 0.034), and a markedly reduced prevalence of depression and apathy [59]. In contrast, a subsequent larger randomized controlled trial evaluating global non-motor outcomes including cognition, mood, and quality of life, did not find statistically significant overall improvements, suggesting that the benefits of GLP-1 agonists may be more domain-specific than generalizable across all non-motor symptoms [32].

More recently, another study assessed the impact of Liraglutide over 54 weeks. While motor and cognitive outcomes did not differ significantly from placebo, there was a notable improvement in total NMSS scores, and activities of daily living improved significantly as reflected in MDS-UPDRS Part II scores [57].

Additionally, real-world data suggest a broader neuroprotective potential, as GLP-1 RAs like semaglutide have been linked to reduced dementia risk in patients with T2D [60]. These findings further support a potential therapeutic role of GLP-1 RAs in targeting specific non-motor domains, particularly those affecting mood, motivation, and functional independence, even when global measures remain unchanged. Previous clinical trials with GLP-1 receptor agonists are outlined in Table 2.

**Table 2:** Summary of GLP-1 in PD clinical trials

Title	Clinical trial ID	Phase	Intervention and dose	Evaluation time points	Findings	Reference
Exenatide and the treatment of patients with Parkinson’s disease	NCT01174810	Phase 2, randomized, single-blind controlled trial	Exenatide subcutaneous, 5 µg daily for 1 month, then 10 µg daily for 11 months	Baseline, 6, 12, and 14 months	Clinically relevant improvements in motor and cognitive outcomes (MDS-UPDRS + 2.7 vs. − 2.2, *p* = 0.037); well tolerated, though weight loss was common.	[54]
Exenatide once weekly versus placebo in Parkinson’s disease	NCT01971242	Phase 2, randomized, double-blind, placebo-controlled, parallel-group, single-center trial	Exenatide subcutaneous, 2 mg once weekly	Baseline, 3, 6, 9, 12, and 15 months	Exenatide improved MDS-UPDRS Part III off-medication scores by 1.0 point (95% CI − 2.6 to 0.7), although the longevity of this effect remains uncertain.	
Evaluation of NLY01 in Parkinson’s disease	NCT04154072	Phase 2, randomized, double-blind, placebo-controlled study	NLY01 (pegylated exenatide) subcutaneous, 2.5 mg or 5.0 mg	Baseline and 9 months	No significant changes in motor or non-motor symptoms were detected compared to placebo.	[57]
Liraglutide improves non-motor function and activities of daily living in Parkinson’s disease	NCT02953665	Phase 2, randomized, double-blind, placebo-controlled trial	Liraglutide 1.2 or 1.8 mg subcutaneous daily, titrated as tolerated	Baseline and 54 weeks (with interim at 28 weeks)	Improved non-motor symptoms (NMSS + 6.6 vs. −6.5 placebo, *p* = 0.07) and activities of daily living (MDS-UPDRS II − 4.1, *p* = 0.001); no significant changes in motor scores or cognition. Common AEs: injection site and GI symptoms.	[56]
Trial of Lixisenatide in early Parkinson’s disease	NCT03439943	Phase 2, investigator-initiated, multicenter, randomized, double-blind, placebo-controlled trial	Lixisenatide subcutaneous, 10 µg daily for 14 days, then 20 µg daily	Baseline, 6, 12, and 14 months	Improvement in MDS-UPDRS III motor scores at 12 months compared to placebo, though gastrointestinal side effects were common. Larger and longer trials are needed to confirm these findings.	[55]
Exenatide once weekly versus placebo as a potential disease-modifying treatment for Parkinson’s disease in the UK	NCT04232969	Phase 3, multicenter, double-blind, placebo-controlled, parallel-group randomized trial	Extended release exenatide 2 mg subcutaneous injection once weekly	Baseline and 96 weeks	No significant difference in MDS-UPDRS part III OFF-medication scores at 96 weeks (adjusted coefficient 0.92, *p* = 0.47). Exenatide was well tolerated, with similar rates of serious adverse events (9% vs. 11% placebo).	[34]

#### Biomarkers and Imaging Correlates

In patients with PD, GLP-1 receptor agonists have demonstrated biologically plausible mechanisms by enhancing brain insulin signaling. Specifically, exenatide treatment over 48 to 60 weeks increased tyrosine phosphorylation of insulin receptor substrate 1 (IRS-1) in neuronal-derived extracellular vesicles (exosomes), alongside activation of downstream signaling proteins such as total Akt and phosphorylated mTOR [61]. These molecular changes correlated with clinical motor improvements, suggesting a potential disease-modifying effect consistent with other trials showing both motor and cognitive benefits as well as biomarker evidence of enhanced insulin and neurotrophic signaling [32, 55].

In contrast, evidence from neuroimaging remains limited. In one early trial, no significant changes in [¹²³I]FP-CIT SPECT uptake were observed over 12 months, likely due to the advanced disease stage of participants [54]. A more recent study using statistical parametric mapping suggested a reduced rate of DaT binding decline in exenatide-treated patients, particularly in the putamen and caudate, but results reached significance only at uncorrected thresholds (*p* = 0.0034 and *p* = 0.0018, respectively) [32]. In another study, 73 of 77 participants underwent repeat DaT–SPECT imaging at 96 weeks, but no significant differences in striatal binding changes were observed between the exenatide and placebo groups across all regions analyzed [34]. Overall, while some data hint at potential neuroprotective effects, current imaging results are inconclusive and highlight the need for further studies in larger, early-stage cohorts where imaging biomarkers are more sensitive to change.

#### Relationship with Clinical Phenotype and Metabolic Comorbidities

Metabolic comorbidities may influence the clinical presentation of PD, leading to an earlier onset of cognitive impairment, a greater burden of axial symptoms, and a reduced response to dopaminergic medication [62]. Emerging evidence from subgroup analyses suggests that patients with PD who have comorbid insulin resistance (established as HbA1C > 39mmol/mol) and obesity (defined as BMI > 25.0) may benefit more from GLP-1 RAs. These benefits include stabilization of cognitive function, although there were no significant differences concerning BMI and HbA1C between responders and non-responders [63]. These findings point towards a stratified, phenotype-based treatment approach. In the UK multicenter randomized placebo-controlled trial, no subgroup stratified by baseline BMI demonstrated a greater likelihood of responding to Exenatide relative to the rest of the study population [34].

## Discussion

This systematic review highlights the multifaceted therapeutic potential of GLP-1 RAs in patients with PD, particularly through their neuroprotective, metabolic, and symptomatic effects. Preclinical evidence consistently demonstrates that GLP-1 RAs can attenuate key pathophysiological mechanisms of PD [40, 53].

Clinical trials, while heterogeneous in design and outcome, broadly support the potential of GLP-1 RAs to improve motor symptoms and possibly delay disease progression. Exenatide and lixisenatide, in particular, have shown consistent motor improvements, with some trials also reporting sustained cognitive benefits and improvements in mood and apathy [32, 54, 56, 59]. However, not all agents or studies have replicated these results, which highlights the importance of considering drug-specific pharmacodynamics, trial design, and patient characteristics when interpreting outcomes [34, 57, 58].

The failure of adipose tissue to expand adequately plays a central role in the development of insulin resistance and T2D. This concept has been reframed through the lens of allostasis, in which there is a progressive overload of metabolic systems in response to chronic nutrient excess. Once the primary visceral (intra-abdominal) fat depots are saturated, free fatty acid spillover into non-adipose tissues and begin to accumulate in ectopic fat depots (liver, skeletal muscle, heart, pancreas, kidney, bone marrow, and central nervous system (CNS) which leads to lipotoxicity, inflammation, and ultimately B-cell dysfunction [64–71]. In the CNS it induces hypothalamic gliosis and lipid accumulation, which may disrupt appetite and energy regulation [67].

GLP-1RA can reduce ectopic fat through appetite suppression and weight loss, improving insulin sensitivity, enhancing fatty acid oxidation, through anti-inflammatory and directly through tissue-specific action via GLP-1 receptors in liver, heart, pancreas and brain [72–75].

A major consideration in the application of GLP-1 RAs to PD is their metabolic impact. Although these agents reliably reduce weight and fat mass in patients with T2D, their effects in normoglycaemic PD populations appear less pronounced [12, 13, 31–34]. This may be due to lower baseline insulin resistance, preserved GLP-1 signaling, or disease-related alterations in appetite regulation [35]. Notably, individuals with PD and coexisting metabolic comorbgidities such as insulin resistance or obesity appear to derive greater clinical and cognitive benefits from GLP-1 RA therapy [62, 63].

In contrast, in older or underweight PD patients who are already vulnerable to malnutrition and frailty, appetite suppression and weight loss remain concerns [16, 17, 22].

GLP-1 RAs promote weight loss primarily by reducing fat mass; however, they may also contribute to loss of lean mass, potentially exacerbating sarcopenia. In older frail adults, bimagrumab, an ActRIIB antagonist, has been explored as a potential strategy to counteract sarcopenia by selectively increasing muscle mass. Clinical trials have demonstrated that the use of Bimagrumab, markedly enhances muscle mass and reduces adiposity in older adults with sarcopenia and patients with T2D and obesity by improving body composition and metabolic profiles [76, 77]. Bimagrumab has not yet been tested in PD but given its anabolic effects and proven benefit in age-related sarcopenia, it represents a theoretically promising intervention to address muscle loss and frailty in PD.

Therefore, assessing body composition in clinical trials of GLP-1 RAs in patients with PD could be critical to understand their impact beyond glycaemic control. Changes in fat and lean mass may influence motor function, frailty, and disease progression. Detailed body composition analysis could help elucidate the metabolic and functional effects of these therapies in this vulnerable population.

## Future Directions and Recommendations

GLP-1 RAs represent a compelling therapeutic option in PD, offering neuroprotective, symptomatic, and metabolic benefits. Their actions on brain insulin signaling, neuroinflammation, oxidative stress, and mitochondrial function likely contribute to improvements in motor and non-motor symptoms, including cognition, mood, and apathy, while maintaining a neutral effect on dyskinesias.

Although well-established metabolic effects are observed in diabetes and obesity, the impact on body weight and dysfunctional adiposity is less marked in normoglycemic PD patients. Emerging evidence suggests that those patients living with insulin resistance or obesity may derive greater clinical benefit, highlighting the importance of a phenotype-based treatment approach.

To refine their therapeutic role, future studies should conduct larger trials with metabolic stratification, exploring biomarkers for predicting treatment response, and evaluating combined interventions involving diet and exercise. These efforts would be vital to advancing GLP-1 RAs from symptomatic relief toward disease modification and personalized treatment in PD patients with metabolic dysfunction.

Several clinical trials have been launched to evaluate the potential disease-modifying effects of GLP-1 RAs in PD. A phase 2 trial (NCT03659682), with Semaglutide (a more potent GLP-1 RA), not yet recruiting, is designed to assess the effects of once-weekly subcutaneous semaglutide (1 mg) on motor symptoms (MDS-UPDRS III in the OFF state), DAT-SPECT imaging, cognition, and non-motor symptoms over 48 months [39].

In animal models, semaglutide reduces food intake and body weight by activating Adcyap1-expressing neurons in the dorsal vagal complex, a key integrator of gut–brain signals that induce a reduction in food intake via both homeostatic and hedonic pathways [78].

These findings point to the dorsal vagal complex as a neuroanatomical substrate for GLP-1R-mediated anorectic effects. Semaglutide also modulates appetite suppression, energy expenditure and satiety signaling through its binding to GLP-1R in the hypothalamus (e.g., arcuate nucleus) [79].

Although GLP-1RAs are generally well tolerated and considered safe drugs, as they gain widespread use for obesity, rare but serious adverse effects such as gastroparesis, bowel obstruction, pancreatitis and non-arteritic anterior ischemic optic neuropathy are emerging. Worsening of diabetic retinopathy seems to be related particularly to rapid glycaemic improvement [80].

An exploratory 12-month follow-up study in patients with PD (NCT03456687) comparing exenatide with placebo has been completed, although its results remain unpublished; only 5 subjects were enrolled, despite the original plan to include 20. Two phase 2 trials are currently active but not recruiting: NCT04305002 evaluates weekly exenatide (2 mg) with disease progression measured by FDG-PET and MDS-UPDRS III, while NCT04269642 is investigating PT320, a sustained-release formulation of exenatide (2.0 or 2.5 mg biweekly), in patients with early PD. This latter study aims to determine the impact on motor progression, supported by clinical and neuroimaging outcomes. These ongoing trials represent important steps toward clarifying the role of GLP-1 RAs as disease-modifying agents in PD and may pave the way for more targeted and effective therapies. Supplementary material Table 1.

Glucose-dependent insulinotropic polypeptide (GIP) and GLP-1 are incretin hormones that regulate insulin secretion in response to nutrient intake, to facilitate glucose tolerance [81]. Tirzepatide, a dual GIPR/GLP-1R agonist approved for T2D and obesity, has shown preclinical neurobiological effects consistent with neuroprotection in models of diabetic neurodegeneration, Alzheimer-type amyloidosis, and Parkinson’s disease; however, results are not uniform across studies and, to date, no tirzepatide trials in PD are registered [82–85].

## Conclusion

While GLP-1 RAs represent an attractive dual-targeted strategy in PD, several knowledge gaps remain unanswered. These include identifying robust biomarkers of response, clarifying the optimal therapeutic window, and establishing long-term efficacy and safety across diverse PD phenotypes. Imaging correlates of neuroprotection remain inconclusive. While some studies have demonstrated changes in insulin signaling pathways and neurotrophic markers, others have failed to detect meaningful alterations in dopaminergic imaging [32, 34, 54, 55, 61]. This discrepancy may reflect the limitations of current imaging modalities or the advanced disease stage of participants in these trials.

The use of GLP-1 RAs may be more effective in PD patients with insulin resistance or T2D, due to overlapping metabolic and neuroinflammatory pathways that may enhance therapeutic responsiveness. In addition, they may promote cognitive resilience, potentially through mechanisms involving reduced neuroinflammation, improved insulin signaling, and enhanced mitochondrial function in the brain. In future trials, cerebrospinal fluid levels of α-synuclein oligomers and pro-inflammatory cytokines such as IL-6 and TNF-α may serve as biomarkers of both neurodegeneration and treatment response in PD, potentially reflecting disease-modifying effects through reduced protein aggregation and neuroinflammation.

Given the growing recognition of the interplay between metabolic dysfunction and neurodegeneration, GLP-1 RAs may offer a novel, dual-acting strategy that not only targets core pathological mechanisms of PD but also addresses systemic comorbidities. Their multifaceted effects and favorable safety profile make them an important and timely target for continued investigation in the quest for disease-modifying therapies in PD.

## Key References

- Flint A, Raben A, Astrup A, Holst JJ. Glucagon-like peptide 1 promotes satiety and suppresses energy intake in Humans. J Clin Invest. 1998;101:515–520.
  - A foundational study establishing the appetite-suppressing and weight-modulating effects of GLP-1 in humans. This paper laid the groundwork for subsequent metabolic and therapeutic applications of GLP-1 RAs and is frequently cited in metabolic research.
- Aviles-Olmos I, Dickson J, Kefalopoulou Z, Djamshidian A, Ell P, Soderlund T, et al. Exenatide and the treatment of patients with Parkinson's disease. J Clin Invest. 2013;123:2730–6.
  - A landmark clinical study reporting sustained motor improvements in Parkinson’s disease patients treated with the GLP-1 receptor agonist exenatide. This was the first Human evidence suggesting disease-modifying potential of incretin-based therapies in neurodegeneration. Recognized as a top 1% most cited article in its field, it has become a foundational reference driving the repurposing of antidiabetic drugs in neurology.
- Aviles-Olmos I, Dickson J, Kefalopoulou Z, Djamshidian A, Kahan J, Ell P, et al. Motor and cognitive advantages persist 12 months after exenatide exposure in Parkinson's disease. J Parkinsons Dis. 2014;4(3):337-44.
  - A pivotal follow-up showing that the motor and cognitive benefits of exenatide persist long after discontinuation. By reinforcing the concept of a durable, possibly neuroprotective effect, this study strengthened the rationale for GLP-1 RAs as disease-modifying candidates in Parkinson’s disease and supported the design of larger randomized trials.
- Meissner WG, Remy P, Giordana C, Maltête D, Derkinderen P, Houéto JL, et al. Trial of Lixisenatide in early Parkinson's disease. N Engl J Med. 2024;390:1176–85.
  - A large, double-blind, placebo-controlled trial testing lixisenatide in early Parkinson’s disease. Although the primary motor outcome was neutral, the study confirmed safety and hinted at benefits in secondary domains, solidifying GLP-1 RAs as a viable therapeutic avenue.
- Athauda D, Maclagan K, Skene SS, Bajwa-Joseph M, Letchford D, Chowdhury K, et al. Exenatide once weekly versus placebo in Parkinson's disease: a randomised, double-blind, placebo-controlled trial. Lancet. 2017;390:1664–75.
  - One of the first large, double-blind, placebo-controlled RCTs of a GLP-1 RA in PD, providing strong clinical evidence for symptomatic benefit. Frequently cited and pivotal in shaping further trials.
- Vijiaratnam N, Girges C, Auld G, McComish R, King A, Skene SS, et al. Exenatide once a week versus placebo as a potential disease-modifying treatment for people with Parkinson's disease in the UK: a phase 3, multicentre, double-blind, parallel-group, randomised, placebo-controlled trial. Lancet. 2025;405:627–36.
  - The latest phase 3 multicenter trial examining long-term effects and confirming or challenging earlier findings. Critical for understanding real-world applicability and guiding future regulatory decisions.

## Supplementary Information

Below is the link to the electronic supplementary material.

Supplementary Material 1

## Data Availability

No datasets were generated or analysed during the current study.

## Abbreviations and Acronyms

AD: Alzheimer Disease
Akt: Protein Kinase B
AMPK: AMP-activated Protein Kinase
BMI: Body Mass Index
BDNF: Brain-Derived Neurotrophic Factor
cAMP: Cyclic Adenosine Monophosphate
CNS: Central Nervous System
CREB: CAMP Response Element-Binding Protein
DATSPECT: Dopamine Transporter Single-Photon Emission Computed Tomography
DRS2: Dementia Rating Scale-2
FDGPET: Fluorodeoxyglucose Positron Emission Tomography
GDNF: Glial Cell-Derived Neurotrophic Factor
GIP: Glucose-dependent Insulinotropic Polypeptide
GIPR: Glucose-dependent Insulinotropic Polypeptide Receptor
GLP1: Glucagon-Like Peptide-1
GLP1RA: Glucagon-Like Peptide-1 Receptor Agonist
HbA1c: Hemoglobin A1c
IL6: Interleukin-6
IRS1: Insulin Receptor Substrate 1
LED: Levodopa Equivalent Dose
MADRS: Montgomery-Åsberg Depression Rating Scale
MDSUPDRS: Movement Disorder Society Unified Parkinson’s Disease Rating Scale
MPPT: 1-Methyl-4-phenyl-1,2,3,6-tetrahydropyridine
mTOR: Mechanistic Target of Rapamycin
NFKB: Nuclear Factor kappa-light-chain-enhancer of Activated B Cells
NMSS: Non-Motor Symptoms Scale
PD: Parkinson’s Disease
PGC1α: Peroxisome Proliferator-Activated Receptor Gamma Coactivator 1-alpha
PI3K: Phosphoinositide 3-Kinase
PT320: Sustained-Release Formulation of Exenatide
ROS: Reactive Oxygen Species
SNpc: Substantia Nigra Pars Compacta
T2D: Type 2 Diabetes
TNFα: Tumor Necrosis Factor Alpha
TrkB: Tropomyosin Receptor Kinase B
UPDRS: Unified Parkinson’s Disease Rating Scale
