# Supplementary material for: Targeting Metabolic Dysfunction in Parkinson’s Disease: The Role of GLP-1 Agonists in Body Weight Regulation and Neuroprotection
Source: Curr Diab Rep. 2025 Sep 26;25(1):49. doi: 10.1007/s11892-025-01606-1 (PMC12474678; doi:10.1007/s11892-025-01606-1)
Supplement: Supplementary file 1 — Supplementary Material 1 [file 11892_2025_1606_MOESM1_ESM.docx]

# **Supplementary material**

# **Table 1. Ongoing Clinical Trials: GLP-1 Receptor Agonists in Parkinson’s Disease**

| **Trial (ID)** | **Phase** | **Status** | **Intervention** | **Main Objective / Notes** |
| --- | --- | --- | --- | --- |
| NCT03659682 | Phase 2 | Not yet recruiting | Semaglutide 1 mg weekly s.c. | Motor function (MDS-UPDRS III OFF) at 48 months, DAT-scan, cognition, non-motor symptoms |
| NCT03456687 | Phase 1 | Completed | Exenatide vs placebo | Exploratory study – no results published yet |
| NCT04305002 | Phase 2 | Active, not recruiting | Exenatide 2 mg weekly | Progression assessed by FDG-PET and MDS-UPDRS III |
| NCT04269642 | Phase 2 | Active, not recruiting | PT320 (sustained-release exenatide) 2 or 2.5 mg | Effect on progression in early PD |

Abbreviations: DAT=Dopamine Transporter; FDG-PET=Fluorodeoxyglucose Positron Emission Tomography, MDS-UPDRS III=Movement Disorder Society-Unified Parkinson’s Disease Rating Scale, Part III; PD=Parkinson’s Disease; s.c.=Subcutaneous.
